# 5’-Terminal AUGs in *Escherichia coli* mRNAs with Shine-Dalgarno Sequences: Identification and Analysis of Their Roles in Non-Canonical Translation Initiation

**DOI:** 10.1371/journal.pone.0160144

**Published:** 2016-07-28

**Authors:** Heather J. Beck, Ian M. C. Fleming, Gary R. Janssen

**Affiliations:** Department of Microbiology, Miami University, Oxford, Ohio, United States of America; University of Lethbridge, CANADA

## Abstract

Analysis of the *Escherichia coli* transcriptome identified a unique subset of messenger RNAs (mRNAs) that contain a conventional untranslated leader and Shine-Dalgarno (SD) sequence upstream of the gene’s start codon while also containing an AUG triplet at the mRNA’s 5’- terminus (5’-uAUG). Fusion of the coding sequence specified by the 5’-terminal putative AUG start codon to a *lacZ* reporter gene, as well as primer extension inhibition assays, reveal that the majority of the 5’-terminal upstream open reading frames (5’-uORFs) tested support some level of *lacZ* translation, indicating that these mRNAs can function both as leaderless and canonical SD-leadered mRNAs. Although some of the uORFs were expressed at low levels, others were expressed at levels close to that of the respective downstream genes and as high as the naturally leaderless *c*I mRNA of bacteriophage λ. These 5’-terminal uORFs potentially encode peptides of varying lengths, but their functions, if any, are unknown. In an effort to determine whether expression from the 5’-terminal uORFs impact expression of the immediately downstream cistron, we examined expression from the downstream coding sequence after mutations were introduced that inhibit efficient 5’-uORF translation. These mutations were found to affect expression from the downstream cistrons to varying degrees, suggesting that some 5’-uORFs may play roles in downstream regulation. Since the 5’-uAUGs found on these conventionally leadered mRNAs can function to bind ribosomes and initiate translation, this indicates that canonical mRNAs containing 5’-uAUGs should be examined for their potential to function also as leaderless mRNAs.

## Introduction

Translation initiation is the rate-limiting step in protein synthesis and requires ribosomes to recognize and bind messenger RNAs (mRNA). In the conventional pathway of initiation, a ternary complex is formed between the 30S ribosomal subunit, the mRNA, and the initiator tRNA with the aid of three initiation factors (for review see [1]). Briefly, 30S ribosomal subunits bind the Shine-Dalgarno (SD) sequence of the mRNA via complementary pairing to the 16S rRNA anti-Shine-Dalgarno (aSD) sequence [2, 3]. The initiator tRNA binds to the complex, stabilized by the SD-aSD interaction, and promotes the proper placement of the start codon in the P-site of the ribosome with subsequent translation initiation [4, 5]. The mechanism for mRNA-ribosome binding via the SD-aSD has been well studied and is thought to be a prerequisite step for translation initiation [1], ([6] and references therein). However, an increasing number of genes have been identified that lack SD sequences or lack a 5’ untranslated region (5’-UTR) altogether [7]. mRNAs lacking a 5’-UTR are referred to as leaderless mRNAs and have been reported in all domains of life [8]. Leaderless mRNA lack ribosome binding signals that would otherwise be contained within the 5’-UTR, such as the SD sequence. The widespread occurrence of these non-canonical mRNAs suggests that they contain features allowing recognition by ribosomes from all translation systems.

Leaderless mRNA appear to follow a novel pathway by which a 70S ribosome binds the 5’-terminal AUG to initiate translation [9, 10]. Binding depends on an AUG initiation codon and is stabilized by initiator tRNA in *Escherichia coli* [11–13]. Addition of a 5’-terminal AUG to an internal segment of *lacZ* mRNA makes it competent to form ternary complexes with 70S ribosomes and initiator tRNA [11], suggesting that a 5’-terminal AUG might be a sufficient signal for ribosomes to identify a leaderless mRNA. Furthermore, a thirty-nucleotide deletion from the 5’-terminus of the naturally occurring leaderless *c*I mRNA results in the loss of the ability to bind ribosomes. However, addition of a 5’-terminal AUG triplet to the truncated *c*I leaderless mRNA restores 70S ribosome binding and allows it to compete with the native leaderless mRNA for ribosome binding *in vitro* and form translationally active complexes *in vivo* [11]. Taken together, these results show that the presence of a 5’-terminal AUG is sufficient for an RNA molecule to bind ribosomes and be translated as a leaderless mRNA.

To further investigate the hypothesis that a 5’-terminal AUG is sufficient for translation of an mRNA, we sought to identify AUG triplets that occur at the 5’-termini of canonical, SD-led mRNAs. Such 5’-terminal AUGs would have the potential to bind ribosomes and allow for translation of a second open reading frame (ORF), in addition to translation from the start codon of the downstream SD-led coding sequence (CDS). Previous work has demonstrated the abundance of small ORFs within 5’-UTRs in both eukaryotic and prokaryotic organisms. In prokaryotic organisms, these small ORFs often encode small peptides [14] and can regulate downstream ORF expression [15, 16]. In this study, we utilized the RegulonDB database [17] to conduct an *in silico* search of *E*. *coli* for canonical, SD-led mRNAs that contain 5’-terminal AUG triplets. These 5’-terminal upstream AUGs (5’-uAUGs) were assayed for their ability to act as initiation codons for translation of the putative 5’-terminal upstream open reading frame (5’-uORF). The 5’-uORF translational activity and ability to bind ribosomes was also analyzed, as well as their effect on the translation efficiency of their respective downstream SD-led CDS. Our results suggest that a number of canonical SD-led mRNAs contain 5’-uAUGs that bind 70S ribosomes and support biologically relevant levels of translation. Our results also suggest that certain 5’-uAUGs and their defined ORFs impact regulation of downstream expression.

## Material and Methods

### Bacterial strains

*E*. *coli* DH5α (New England Biolabs [NEB]) was used as the host for all plasmid DNA manipulations. *E*. *coli* RFS859 (F-, *thr*-1, *ara*C859, *leu*B6, *Dlac*74, *tsx*-274, l-, *gyrA*111, *recA*11, *relA*1, *thi*-1) [18] was used as host for the expression and assay of *lacZ* fusion mRNA constructs. *E*. *coli* K12 total genomic DNA was used as a template for PCR amplifications to isolate the genes of interest used in this study.

### Reagents

Radio-labeled [γ-32P] ATP (6000 Ci/mmol, 150 mCi/mL) was purchased from Perkin Elmer. Restriction endonucleases, T4 DNA ligase, T4 polynucleotide kinase (PNK), and T7 RNA polymerase were purchased from New England Biolabs and used according to manufacturer’s instructions. RNase-free DNase I (Roche), AMV reverse transcriptase (Life Sciences), and Pfu DNA polymerase (Stratagene) were used according to the manufacturer’s specifications. DNA oligonucleotides were purchased commercially (IDT). The *lacZ*-specific oligonucleotide 5’-GTTTTCCCAGTCACGACGTTG-3’, which anneals to positions +99 to +78 of the *lacZ* coding sequence in *lacZ* fusions, was used in the primer extension inhibition (toeprint) assays.

### Construction of *lacZ* fusions

Codons 1–16 of each gene tested, including the putative upstream open reading frame (uORF) expressed within 5’-UTR, were fused to the fifth codon of a *lacZ* reporter gene and cloned into pUC18-derivative plasmids [19] containing an ampicillin resistance marker. The constructs contained an upstream *lac* promoter (TATAAT). The 5’-uORF for each gene tested was fused to the fifth codon of a *lacZ* reporter gene just upstream of its in-frame stop codon.

### β-galactosidase assay

β -galactosidase assays were performed as previously described [20].

### Preparation of *in vitro* synthesized transcripts

The cloned plasmids were used as templates in PCR amplifications utilizing a primer to incorporate the T7 RNA polymerase promoter sequence (5’-TAATACGACTCACTATAG-3’). This produced DNA fragments containing a T7 promoter sequence, allowing for *in vitro* transcription with T7 RNA polymerase and production of RNA used in toeprint reactions. RNAs were synthesized and purified as described [21]. RNAs used in toeprint assays were synthesized by combining purified PCR amplicons (constructs with *lacZ* fusions containing a T7 promoter) and T7 RNA polymerase in 1X buffer (40 mM Tris-HCl at pH 7.8, 25 mM MgCl_2_, 1 mM spermidine, 0.01% Triton X-100, 5 mM each NTP, and 30 mM dithiothreitol). Transcription reactions were incubated for approximately 4 h at 37°C, and 40 mM ethylenediamineetetraacetic acid (EDTA) was added. Samples were treated with DNase (Roche) for 15 min at 37°C. RNA was ethanol-precipitated and suspended in RNA loading dye (50% formamide, 0.05% bromophenol blue, 0.05% xylene cyanol). Samples were subjected to polyacrylamide gel electrophoresis (PAGE; 6% acrylamide, 7 M urea) and full-length products were excised using UV shadowing. Gel slices were incubated overnight at room temperature in elution buffer [(300 mM NaOAc at pH 5.2, 0.1% sodium dodecyl sulfate (SDS), 1 mM EDTA)] with gentle rocking. The supernatant was phenol-extracted and ethanol-precipitated.

### Ribosome isolation

Isolation of *E*. *coli* MRE600 70S ribosomes and 30S ribosomal subunits was performed as previously described [21]. The same batch of ribosome preparations was used in each initial and duplicate primer extension inhibition assay. For confirmation of unexpected binding signals, a second ribosomal batch preparation was used and in each case the binding signals were reproduced (not shown).

### Primer extension inhibition (toeprint) assay

DNA oligonucleotides were phosphorylated at the 5’-terminus using [γ-32P] ATP (6000 Ci/mmol, 150 mCi/mL; Perkin Elmer) and T4 PNK in 1X kinase buffer for 30 min at 37°C and annealed to 3’-termini of RNA as previously described [22]. Annealed RNA was incubated with 30S subunits or 70S ribosomes with or without tRNA^fMet^ for 15 min at 37°C. Reactions were transferred to ice, and reverse transcriptase was added to extend from the labelled oligonucleotide primer to produce cDNA. The reactions were incubated for 15 min at 37°C and stopped by the addition of 0.3M NaOAc and 100% ethanol and precipitated overnight at -80°C. Precipitated complexes were collected by centrifugation and dissolved in loading dye (80% deionized formamide, 10 mM NaOH, 1 mM EDTA, 0.5% bromophenol blue, and xylene cyanol), followed by heat treatment (95°C, 5 min) and PAGE (6% acrylamide, 7 M urea) in 1X TBE. Gels were visualized via autoradiography. In each case, toeprint assays were performed at least twice with reproducible results.

## Results

### 5’-uORFs support translation

Bioinformatic analysis of the *E*. *coli* RegulonDB transcriptome database [17] of all promoter types identified several canonical mRNAs with an untranslated leader and SD-led open reading frame (ORF) that also contained an AUG triplet within three nucleotides of the mRNA’s 5’-terminus (i.e., 5’-AUG, NAUG, NNAUG, NNNAUG) [23]. Of the 3,456 *E*. *coli* transcripts in RegulonDB, 115 transcripts have an AUG at the experimentally demonstrated or predicted 5’-terminus and 287 transcripts have an AUG within three nucleotides of their 5’-terminus (S1 Table). In addition to undergoing translation as canonical SD-led mRNAs from an internal start codon, we predicted that these mRNAs might also be translated as leaderless mRNAs from AUG triplets located at, or near, their 5’-termini, thereby categorizing them as bicistronic mRNA. We selected a subset of thirteen genes for further study, chosen on the basis of characteristics that include the predicted gene’s function, length of the putative peptide encoded from the 5’-uORF, the distance of the 5’-uAUG triplet from the 5’-terminus, and the position of the uORF’s stop codon relative to the downstream ORF’s start codon (Table 1). We chose genes in which the 5’-uAUG was out of frame with the downstream start codon to select bicistronic mRNAs rather than genes in which translation of the 5’-uORF could produce longer isoforms of the canonical gene product.

**Table 1:** Selected genes examined that contain an AUG triplet at the 5’-terminus of its transcribed canonical mRNA.

Gene	Function	Length of putative peptide	Distance from 5’-terminal	Distance from uORF stop to ORF start
*cmk*	Cytidylate kinase	7 a.a.	2 nt	10 nt
*fucP*	L-fucose transporter	12 a.a.	0 nt	77 nt
*glpF*	Glycerol uptake facilitator	17 a.a.	0 nt	17 nt
*iscR*	Iron sulfur cluster DNA-binding transcriptional repressor	18 a.a.	2 nt	8 nt
*luxS*	S-ribosylhomocysteinase	26 a.a.	2 nt	0 nt
*mngR*	DNA-binding transcriptional dual regulator, fatty-acyl-binding	15 a.a.	1 nt	2 nt
*pcnB*	Poly(A)polymerase I	14 a.a.	0 nt	17 nt overlap
*pnp*	Polynucleotide phosphorylase	22 a.a.	0 nt	89 nt
*ptrB*	Protease II	21 a.a.	0 nt	34 nt overlap
*rcnR*	Transcriptional repressor	11 a.a.	0 nt	86 nt
*rhaB*	Rhamnulose kinase	8 a.a.	1 nt	ATGA overlap
*uvrY*	Response regulator	5 a.a.	1 nt	28 nt
*xap*	DNA binding transcriptional activator	18 a.a.	0 nt	26 nt overlap

An overlap refers to a uORF stop codon that is within the downstream coding sequence, albeit out of frame.

**Fig 1:**
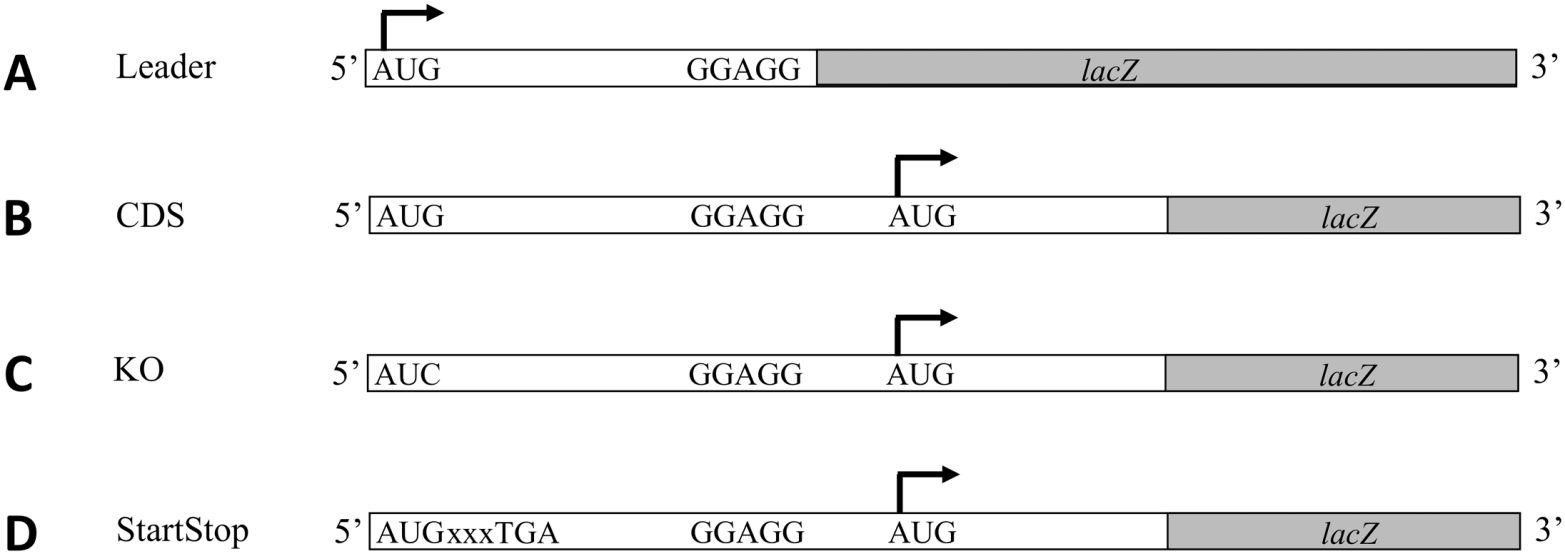
Schematic of *lacZ* fusions constructed for each gene tested. *lacZ* fusions to RNA encoding the 5’-uORF (A) or to the coding sequence (CDS) of the downstream gene (B-D). The 5’-uAUG is either maintained as AUG (CDS), mutated to AUC (KO) or contains a premature stop codon (StartStop). The arrow indicates the in-frame AUG start codon defining the ORF providing expression of the *lacZ* reporter gene.

The translational activity of each of the 5’-uORFs was assessed by an in-frame fusion to a *lacZ* reporter gene and transcribed from the *lac* promoter (Fig 1A). β-galactosidase assays [20] were performed to measure translation from the 5’-uORFs. Activity measured from the products of *lacZ* fusions to naturally leaderless *E*. *coli* bacteriophage λ *c*I [24] and transposable element *Tn*1741 *tetR* [25] mRNAs were used as controls for comparison of expression levels.

**Fig 2:**
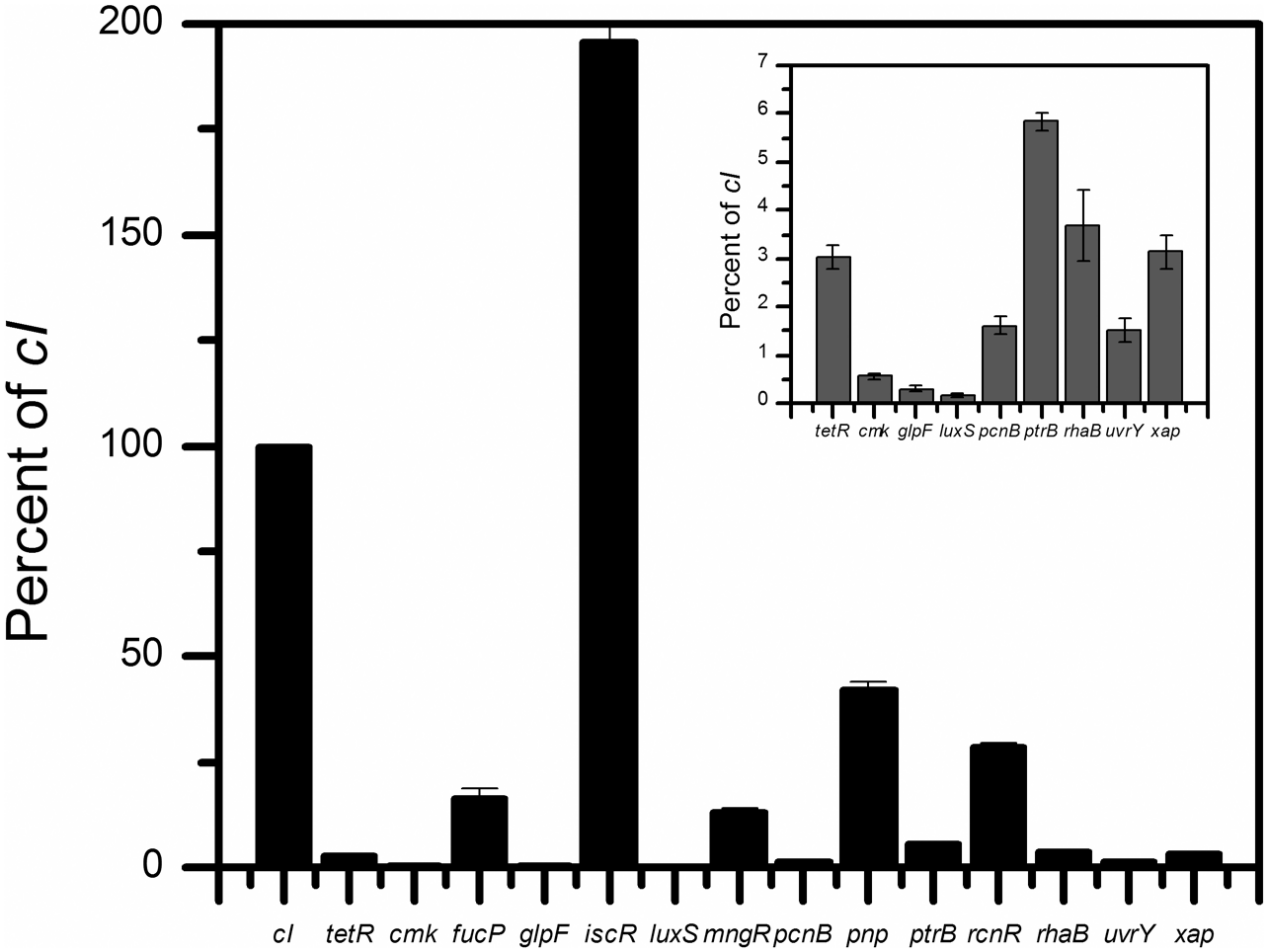
5’uAUGs support varying levels of translation compared to known leaderless mRNA in *E*. *coli*. Translation from select 5’-uORFs fused to *lacZ* (see Fig 1A) using β-galactosidase assays performed in triplicate. Translation is compared to β-galactosidase activity measured from *cI-lacZ* (= 100%) fusions. A subset of 5’-uORFs displaying lower levels of translation are shown as compared to β-galactosidase activity measured from *cI-lacZ* (= 100%) fusions (inset).

Varying levels of expression were seen from the 5’-uORFs, with many falling within the expression range measured for the *tetR* and *c*I leaderless mRNAs (Fig 2). *cmk*, *glpF*, and *luxS* 5’-uORFs were expressed at levels 10-fold lower than *tetR*, whereas *uvrY* and *pncB* 5’-uORFs were expressed at levels only 2-fold lower than *tetR* (Fig 2, inset). Conversely, *ptrB*, *rhaB* and *xap* 5’-uORFs were expressed at levels very similar to *tetR* (Fig 2, inset). Several 5’-uORFs were actually expressed at levels 2-fold to 10-fold higher than *tetR* (e.g., *mngR*, *fucP*, *rcnR* and *pnp*) (Fig 2), albeit still significantly lower than *cI* (Fig 2). The most highly expressed 5’-uORF tested was *iscR*, which was expressed at a level 2-fold greater than *cI* (Fig 2). These results show that the 5’-uAUGs can act as an initiation codon to support translational expression, thereby identifying previously unexplored ORFs.

*lacZ* fusions were also constructed to assess the translational activity of the canonical SD-led downstream CDS (Fig 1B). In each instance, the 5’-uORF and the downstream SD-led ORF are in different reading frames or the uORF’s stop codon is upstream of the annotated ORF and could therefore be compared individually via LacZ fusions. This is important to note because in some instances the two cistrons overlap but will still produce two different gene products (Table 1). Only one gene tested, *rcnR*, supported equal levels of expression from both ORFs (Table 2). However, three genes, *pnp*, *iscR*, and *mngR*, had 5’-uORFs that were translated at levels ranging from 10–50% of downstream CDS expression (Table 2). The majority of 5’-uORFs tested were not expressed at comparable levels (Table 2). There is large variation in the levels of translation from these 5’-uAUGs and the basis for these differences is still unclear. These results demonstrate that an AUG codon at the 5’-terminus of an ORF in the untranslated region of a canonical, SD-led mRNA has the potential to function as a start codon and support significant translational activity. However, the presence of a 5’-uAUG is not always sufficient for translation. Comparable expression to known leaderless mRNAs, as well as to their downstream cognate CDS in some cases, suggests the potential importance of 5’-uAUGs.

**Table 2:** Translation from the 5’-uORF as a percent of translation relative to its downstream canonical CDS as determined by β-galactosidase assays performed in triplicate.

Gene	Translation of 5’-uORF as a percent of downstream CDS
*cmk*	0.3
*fucP*	1.9
*glpF*	0.09
*iscR*	54.0
*luxS*	0.1
*mngR*	10.2
*pcnB*	7.7
*pnp*	28.6
*ptrB*	1.3
*rcnR*	99.7
*rhaB*	2.2
*uvrY*	0.2
*xap*	5.3

### 5’-uAUGs bind 70S ribosomes

**Fig 3:**
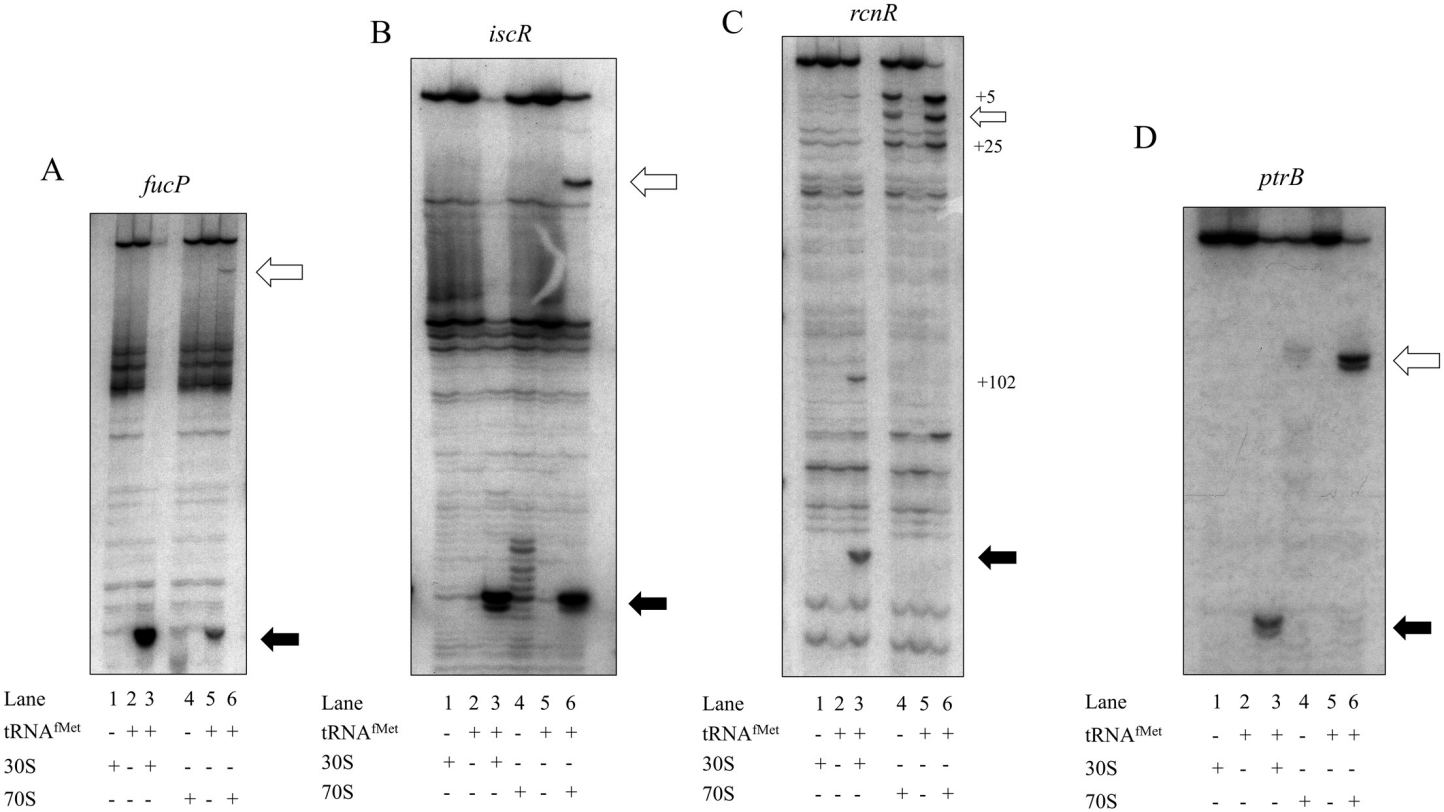
5’-uAUGs bind 70S ribosomes following the proposed leaderless mRNA initiation mechanism. Primer extension inhibition (toeprint) reactions contain mRNA (A) *fucP*, (B) *iscR* (C) *rcnR* (D) *ptrB*, as well as initiator tRNA, and 30S subunits or 70S ribosomes as indicated by + or—symbols; reaction products are separated on denaturing polyacrylamide gels and visualized by autoradiography. Predicted position of toeprint signals (+15) to the 5’-uAUG (open arrow) and downstream AUG (closed arrow) are indicated. Additional positions displaying ribosome dependent bands are also indicated. Figure is representative of experiments performed in biological triplicate.

Primer extension inhibition (toeprint) assays were performed to assess ribosome binding patterns to the 5’-uAUG start codons and the internal SD-led start codons. *In vitro* transcribed mRNAs, corresponding to 5’-uORFs exhibiting varying expression levels (*fucP*, *iscR*, *rcnR*, and *ptrB*) (Fig 2), were tested by toeprint assays to analyze the inherent affinity of ribosome complexes for the 5’-uAUG. Assays were performed in the presence of initiator tRNA and either 30S ribosomal subunits or intact 70S ribosomes. As expected, 70S ribosomes bound the 5’-uAUGs, whereas 30S subunits bound the internal CDS start codon for each gene tested (Fig 3). This ribosome binding pattern suggests that these mRNAs interact with ribosomes both as canonical leadered mRNAs, with 30S subunits binding to the CDS start codon, and as leaderless mRNAs with 70S ribosomes binding to the 5’-uAUGs. This further supports the notion that the 5’-uAUGs are functioning as initiation codons for uORF translation.

In the case of *fucP*, a 70S toeprint signal was observed at the 5’-uAUG which displays weak intensity compared to the downstream 30S subunit signal. The relationship between the two signals correlates with the *fucP* expression data (Table 2). The presence of a 70S toeprint signal at the 5’-uAUG confirms the ability of the *fucP* 5’-uORF to bind ribosomes (Fig 3A) and be expressed as a leaderless mRNA (Fig 2). The *fucP* downstream CDS was shown to be relatively highly expressed (Table 3), which agrees with the strong internal toeprint signals. Interestingly, 70S ribosomes also appeared to bind *fucP*’s internal AUG *in vitro*. The internal 70S ribosome binding phenomenon was also seen in the case of *iscR* mRNA.

**Table 3:** Expression levels from downstream CDS fusions to *lacZ* that contain an intact 5’-uAUG (CDS), a mutated 5’-uAUC (KO), or an intact 5’-AUG with a premature stop codon (StartStop) (see Fig 1) including the standard deviation from triplicate cultures.

Gene	CDS	KO (% difference from CDS fusion)	StartStop (% difference from CDS fusion)
*cmk*	9.8 ±0.3	11.5±1.4 (+17%)	11.3±0.4 (+15%)
*fucP*	6.8±1.0	5.7±0.2 (-17%)	4.5±0.06 (-35%)
*glpF*	13.0±2.4	10.6±2.9 (-18%)	NA
*iscR*	26.6±1.7	35.3±1.4 (+33%)	NA
*luxS*	11.3±0.8	13.3±1.3 (+18%)	7.0±0.3 (-37%)
*mngR*	9.2±0.7	14.0±1.3 (+52%)	5.0±0.3 (-45%)
*pcnB*	3.6±0.6	3.9±0.4 (+10%)	NA
*pnp*	28.6±0.7	25.5±0.2 (-11%)	NA
*ptrB*	5.6±0.8	0.4±0.02 (-93%)	2.3±0.1 (-59%)
*rcnR*	4.4±0.6	1.4±0.4 (-69%)	NA
*rhaB*	7.7±0.1	8.0±0.6 (+4%)	13.5±2.4 (+76%)
*uvrY*	47.2±2.5	46.2±2.4 (-3%)	NA
*xap*	10.6±0.2	10.3±0.6 (0%)	NA

Expression levels are expressed in thousands of Miller Units. The expression as a percent difference from the downstream CDS fusion to *lacZ* is included in parenthesis. NA = not available.

30S subunits bound to the SD-led CDS start codon of *iscR* mRNA and 70S ribosomes bound to the 5’-uAUG (Fig 3B), as expected. 70S ribosomes binding to *iscR* mRNA’s 5’-uAUG (Fig 3B) corresponds with a high level of *iscR* 5’-uORF expression compared to leaderless *cI* mRNA (Fig 2). In addition, 70S ribosome binding to the internal SD-led start codon of *iscR* was observed, as with *fucP* above. The toeprint signal strength of 70S ribosome binding of the SD-led start codon was nearly as strong as that of the 30S subunit binding to the internal start codon. There have been other reports of internal 70S ribosome binding in toeprint assays, but the 70S ribosome binding is typically weaker than 30S subunit binding at the internal position [9, 26]. It is interesting that internal 70S ribosomal binding was seen for both *fucP* and *iscR*, although not for *rcnR* or *ptrB* (Fig 3), suggesting that, for reasons that are still unclear, certain mRNAs have features that allow 70S internal binding *in vitro*. It is possible that 70S internal binding is always present, but less stable for certain mRNAs although further investigation must be completed to examine this line of inquiry. The observation that 70S ribosomes did not bind the CDS of all tested transcripts (Fig 3) demonstrates the absence of contamination of 30S subunits and absence of ribosomal splitting occurring within the 70S ribosomal preparations. Assays reproduced with different 70S batch preparations and different mRNA preparations consistently gave the same results (data not shown).

As expected, 30S subunits bound to the SD-led CDS start codon of *rcnR* mRNA and 70S ribosomes bound to the 5’-uAUG (Fig 3C). The toeprint signal strength for 70S ribosomes bound to the 5’-uAUG of *rcnR* was equal to or greater than that of 30S subunits binding to the internal SD-led AUG. This indicates similar toeprint signal strength, and may reflect the observation that translation from *lacZ* fusions to the *rcnR* 5’-uORF and the downstream SD-led CDS were equivalent (Table 2). The 70S ribosomes binding at the 5’-uAUG, with or without initiator tRNA, displayed multiple toeprint signals at the expected +16 position, but also at the +5 position and the +25 position (Fig 3C lanes 4 and 6). These signals are ribosome-dependent, indicating that they are not the product of secondary structure. Ribosomes appear to stably bind the 5’-terminus without the AUG start codon positioned in the P-site, demonstrating that tRNA codon-anticodon pairing is unnecessary, supporting tRNA-independent binding. Although this signal pattern has been reproduced in subsequent toeprint assays (data not shown), further experimentation is necessary to explore if this ribosome binding pattern reflects the process of ribosomal loading on 5’-terminal AUGs.

Aside from the predicted 30S subunit toeprint binding signal binding to the downstream *rcnR* CDS start codon, there was another tRNA-dependent signal at position +102 corresponding to 30S subunit binding to an AUG at position +88 within the 5’-UTR (Fig 3C lane 3, S2 Table). This potential start codon is out of frame with the *rcnR* CDS start codon and specifies an uORF that would produce a 22-amino acid long putative peptide before encountering a stop codon. These results indicate that there are additional ORFs within the *rcnR* mRNA that may represent additional peptide production.

Similarly, to the other mRNAs tested, 30S subunits bound to the internal SD-led start codon of *ptrB* mRNA, and 70S ribosomes bound to the 5’-uAUG. In the case of *ptrB*, however, the 70S toeprint signal intensity at the 5’-uAUG was not a reliable predictor of expression level. *ptrB*’s 5’-uAUG showed very strong 70S binding as well as tRNA-independent 70S binding (Fig 3D), but expression from the 5’-uORF was low compared to either its downstream CDS (Table 2) or known leaderless mRNA (Fig 2). In this case, the ribosome binding data does not correlate to translational activity seen in β-galactosidase assays which may indicate that select 5’-uAUGs are bound by ribosomes for purposes other than translation initiation.

### 5’-uAUGs can influence downstream gene expression

The 70S toeprint signal intensity of *ptrB* led us to suspect that 5’-uAUGs function not only in peptide production but also as regulatory features. Genes in the same transcriptional unit, such as in an operon, typically have related functions, and in some cases the uORFs can affect expression of downstream cistrons. One example of this type of regulation is referred to as translational coupling [27] and is typically necessary due to sequestration of the downstream ribosome binding regions by secondary structure [28] and is most efficient when the upstream stop codon is in close proximity to the downstream start codon [29]. To assess effects on downstream CDS expression that might result from disruption of 5’-uORF translation, *lacZ* was fused to the SD-led CDS in the presence of a 5’-uAUG knockout mutation (i.e., uAUG→AUC) (Fig 1C). Mutation of the 5’-terminal start codon would be expected to prevent ribosomal binding as well as prevent translation of the 5’-uORF. Mutation of a subset of genes’ 5’-uAUGs to AUCs affected expression of the SD-led downstream CDS to varying degrees (Table 3). Many remained minimally affected, whereas some increased in expression (e.g., *cmk*, *iscR*, *luxS* and *mngR*) and others decreased in expression (e.g., *fucP*, *glpF*, *ptrB*, and *rcnR*) (Table 3).

To determine whether the change in downstream CDS expression was due to loss of translation of the 5’-uORF or loss of ribosome binding to the mRNAs’ 5’-terminus, we made a separate start-stop mutation to introduce a premature stop codon two codons after the 5’-uAUG start codon (i.e., AUGxxxUGA) in a subset of genes (Fig 1D). The 5’-uAUGs may stabilize the mRNA due to their inherent ability to bind ribosomes and can protect the mRNA from degradation by RNases [30]. This protection may contribute to the positive effects the 5’-uAUG has on downstream expression, independent of 5’-uORF translation. In some cases, the start-stop mutation produced results similar to those of the corresponding 5’-uAUG knockout mutations, emphasizing the importance of translation of the 5’-uORF (Table 3). The *luxS* and *mngR* mRNAs both had increased expression levels in the knockout mutant and decreased expression levels in the start-stop mutant (Table 3), highlighting the negative effect of the 5’-uAUG codon on CDS expression.

**Fig 4:**
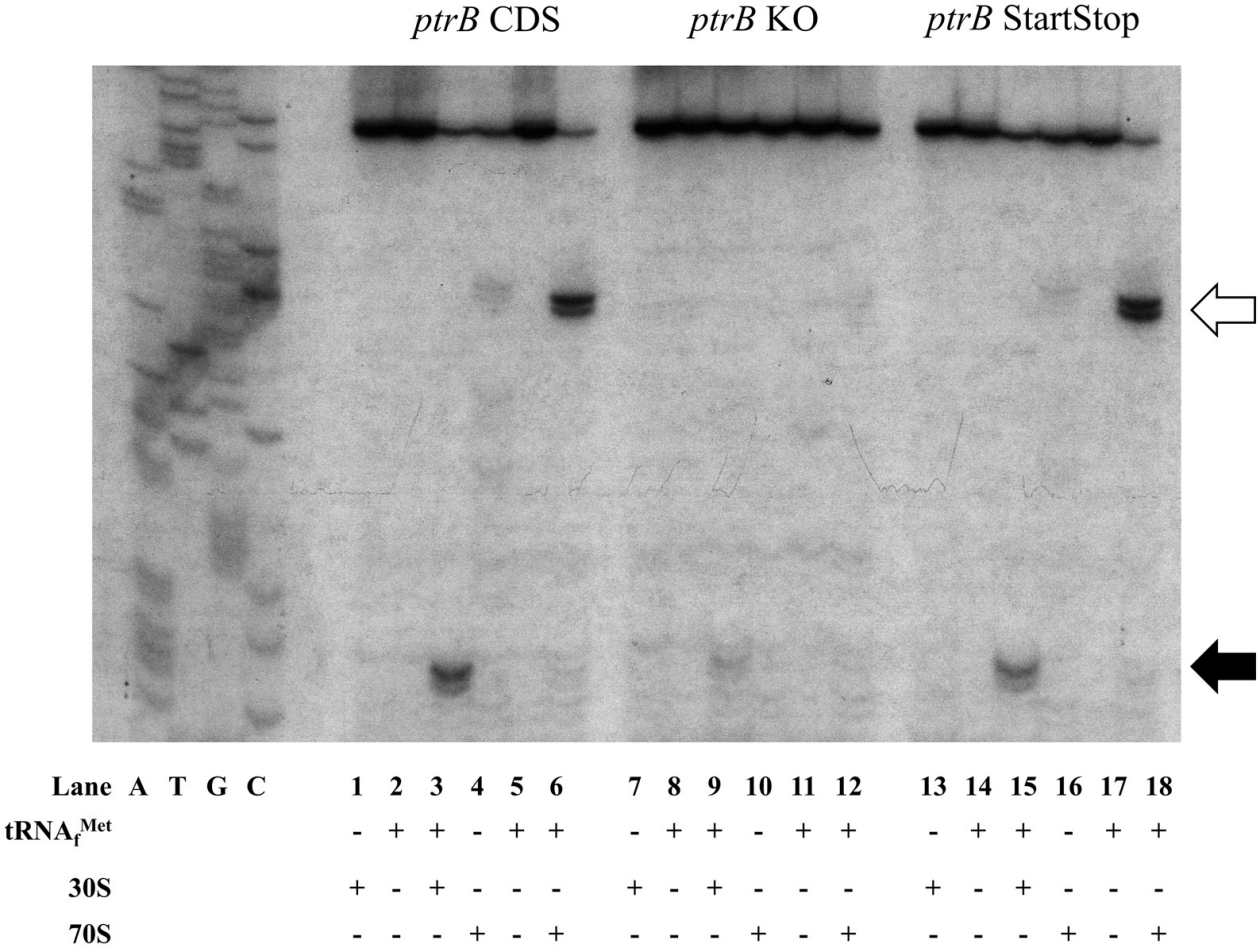
Loss of 30S subunit binding to CDS start codon as a result of the 5’-uAUG mutation. Primer extension inhibition (toeprint) reactions contain mRNA, initiator tRNA, and 30S subunits or 70S ribosomes as indicated by + or—symbols; reaction products are separated on denaturing polyacrylamide gels and visualized by autoradiography. Predicted position of toeprint signals (+15) to the 5’-AUG (open arrow) and downstream AUG (closed arrow) are indicated. *ptrB* sequencing ladder is included on the left. Figure is representative of experiments performed in biological triplicate.

Of the thirteen different genes assayed containing 5’-uAUG→5’-AUC mutations, the *ptrB* gene was particularly affected by the 5’-uAUG mutations. As a result of the 5’-uAUG →5’-AUC mutation, expression from the downstream *ptrB* CDS was drastically reduced to less than 8% (Table 3). Toeprint assays revealed that 30S subunit binding to the downstream start codon in the presence of the 5’-AUC mutation was nearly eliminated (Fig 4), correlating with the expression data (Table 3). However, in the presence of the start-stop mutation, expression was restored to 40% of the wild-type level (Table 3) and reappearance of a 30S subunit toeprint was observed (Fig 4). These data show that *ptrB’s* 5’-uAUG influences 30S subunit binding to the downstream start codon and concomitantly influences expression. This suggests there is a potential regulatory function of *ptrB’s* 5’-uAUG on downstream expression that is not dependent on uORF translation.

## Discussion

The results presented here indicate that AUG triplets at the 5’-termini of SD-led mRNAs can function as start codons and have the ability to bind ribosomes and be translated as leaderless mRNAs. Using β-galactosidase and toeprint assays, we have shown that many 5’-uORFs were translated at biologically relevant levels and bound by 70S ribosomes, with some appearing to play a role in expression of the downstream CDS.

A number of the previously uncharacterized 5’-uORFs supported levels of expression comparable to known leaderless mRNAs or their respective downstream CDS. *rcnR* is one example of an mRNA this study has identified as an efficiently translated bicistronic mRNA. Stable ribosome binding at the 5’-terminus supports high expression levels observed from *rcnR*’s 5’-uORF. Since *rcnR*’s 5’-uORF and CDS expression were similar (Table 2), this suggests nearly equivalent amounts of polypeptide product are being made for each cistron. This is surprising because leaderless mRNA is typically thought to be less efficiently translated than leadered mRNA [31]. Since the *rcnR* 5’-uAUG→AUC mutation reduced downstream CDS expression (Table 3), this 5’-uAUG may also play a role in downstream expression, possibly through translational coupling. Therefore, *rcnR* is an example of a 5’-uAUG that specifies a highly expressed uORF whose expression is linked to downstream expression.

Additional regulatory features, possibly independent of the putative translated peptide, may be present within the 5’-uORF that could influence expression efficiency or function of the downstream CDS. This form of regulation is widespread and has been seen in both prokaryotic [32–35] and eukaryotic systems ([36] and references therein); [37, 38]. Mutation of the 5’-uAUG to AUC impacted downstream expression in *iscR*, *mngR*, *ptrB*, and *rcnR* mRNA (Table 3). In each case, this suggests that disruption of ribosome binding and/or 5’-uORF translation has an effect on the downstream CDS. Conversely, in some mRNAs (i.e., *rhaB*, *uvrY*, and *xap*), no change was seen in CDS expression as a result of the 5’-uAUG mutation (Table 3) indicating that the CDS is expressed independently from any 5’-uORF ribosome binding or translation. This reinforces the idea that 5’-uAUGs have diverse functions, and may function differently in different contexts.

The *ptrB* CDS showed a dramatic decrease in expression, as well as loss of internal 30S subunit binding, in the presence of the 5’-uAUG knockout (Table 3, Fig 4). The dependency on the 5’-uAUG, but not translation of the putative peptide, for ribosome binding and expression of the *ptrB* CDS, suggests regulatory roles. Since the *ptrB* 5’-UTR is only 26 nucleotides long (S2 Table), the regulatory effects may be related to an overlap in ribosomal occupancy causing a steric hindrance and prohibiting both 70S ribosomes and 30S subunits from being bound at the same time. We propose that the 5’-uAUG is the major signal for ribosome recruitment and binding to the mRNA for CDS expression, although the mechanism remains unclear. In one possible scenario, rather than translating the 5’-uORF, this region may act as a standby site [39] for ribosome loading onto the mRNA, taking advantage of inherent binding strength to 5’-AUGs. Once the ribosome is bound to the 5’-terminus, it could then access and bind the downstream CDS translation initiation region. This pre-loading may result in more stable ribosome binding and more efficient internal CDS translation. Further investigation into this potential model may elucidate a novel mechanism of initiation or regulation of translation via a 5’-uAUG.

While the majority of the 5’-uAUGs we tested function in either 5’-uORF translational expression or regulation, some 5’-uAUGs showed no obvious role in these activities. The 5’-uORFs of *cmk*, *glpF*, and *luxS* mRNA were translated at levels much lower than *tetR* (Fig 2), and do not appear to have a substantial effect on downstream CDS expression (Table 3). It is possible that these 5’-uAUGs may have formed by chance and have not been evolutionarily selected for a regulatory role. Alternatively, it is possible that there is an unknown function for these 5’-uAUGs that we have not considered or tested.

Overall, the 5’-uAUGs we tested may possess a variety of predicted functions, such as providing protection to stabilize the mRNA transcript via ribosome binding, producing peptides, and contributing to regulation of the downstream CDS. The ribosome binding studies revealed the translation potential for unannotated uORFs, which may be more widespread than previously thought. Ribosome binding studies similar to the work we have performed could also reveal ORFs that are not detected by visual inspection or bioinformatic analysis. This study provides insight into pitfalls of our current methods of identifying translation initiation sites, and imply that *in silico* analyses may be biased by imposing size limitations and overlooking uORFs when analyzing genomes. The 5’-uORFs may represent an additional subtype of cistron that should continue to be considered when annotating genomes because this study shows their potential to be functional at biologically relevant levels.

## Supporting Information

S1 Table RegulonDB identified genes. Complete list of genes identified by *in silico* analysis of the RegulonDB *E*. *coli* transcriptome as having a Shine-Dalgarno sequence within their 5’-UTR as well as an AUG triplet within three nucleotides of the 5’-terminal (i.e., 5’-AUG, NAUG, NNAUG, and NNNAUG). The nucleotides defined as the 5’-uAUG for each gene are underlined. The table includes the gene name, the first 21 nucleotides at the 5’-terminal end of the mRNA, its start position on the *E*. *coli* chromosome, strand of location, accession number (ECK#), and the sigma factor associated with its transcription.
(XLSX)

S2 Table Sequences of mRNAs tested. List of mRNAs tested in this study and their sequences including their 5’UTRs (lower case) and the first 15 codons of the coding sequences (upper case). The 5’-uAUGs and their in-frame stop codons are upper case and bold. The underlined sequences correspond to the additional *rcnR* putative uORF identified using toeprint assays.
(DOCX)
