# Supplementary material for: 5’-Terminal AUGs in Escherichia coli mRNAs with Shine-Dalgarno Sequences: Identification and Analysis of Their Roles in Non-Canonical Translation Initiation
Source: PLoS One. 2016 Jul 28;11(7):e0160144. doi: 10.1371/journal.pone.0160144 (PMC4965119; doi:10.1371/journal.pone.0160144)
Supplement: S2 Table — List of mRNAs tested in this study and their sequences including their 5’UTRs (lower case) and the first 15 codons of the coding sequences (upper case). The 5’-uAUGs and their in-frame stop codons are upper case and bold. The underlined sequences correspond to the additional rcnR putative uORF identified using toeprint assays. (DOCX) [file pone.0160144.s002.docx]

| mRNA | mRNA sequence |
| --- | --- |
| *cmk* | TT**ATG**ttaacggtacgcctgttt**TAA**ggagataaagATGACGGCAATTGCCCCGGTTATTACCATTGATGGCCCAAGCGGT |
| *fucP* | **ATG**agttcatttcagacaggcaaatattcactgata**TGA**agcccgaactcgctggttttgcacttttgaaaacataaccgattacgtgcttaagcttctgaacctaagaggatgctATGGGAAACACATCAATACAAACGCAGAGTTACCGTGCGGTAGAT |
| *glpF* | **ATG**cctacaagcatcgtggaggtccgtgactttcacgcatacaacaaacat**TAA**ctcttcaggatccgattATGAGTCAAACATCAACCTTGAAAGGCCAGTGCATTGCTGAATTC |
| *iscR* | CT**ATG**caatacccccacttttacaataaaaaaccccgggcaggggcgagtttgagg**TGA**agtaagacATGAGACTGACATCTAAAGGGCGCTATGCCGTGACCGCAATGCTT |
| *luxS* | AA**ATG**cgcgtctttcatatactcagactcgcctgggaagaaagagttcagaaaatttttaaaaaaattaccggaggtggc**TAA**ATGCCGTTGTTAGATAGCTTCACAGTCGATCATACCCGGATGGAA |
| *mngR* | G**ATG**aatttatcgggcataatggttaaattcgtattaatgagatac**TAA**aaATGGGACACAAGCCCTTATACCGGCAGATTGCCGATCGCATTCGT |
| *pcnB* | **ATG**tttgacactaccgaggtgtactATTTTTACCCGAGTCGC**TAA**TTTTTGCCGCAAGGTGCTAAGCCGC |
| *pnp* | **ATG**aatgatcttccgttgcagaggttcgcgcggctaatgagaggctttacccacatagagctgggt**TAG**ggttgtcattagtcgcgaggatgcgcagaagatcgggtattaacaccagtgccgtaaggtactgtctaagaaagagaaaggatattacaTTGCTTAATCCGATCGTTCGTAAATTCCAGTACGGCCAACACACC |
| *ptrB* | **ATG**tttcaaccagaaagaacaataacATGCTACCAAAAGCCGCCCGCATTCCCCACGCCA**TGA**CGCTTCATGGC |
| *rcnR* | **ATG**ataataattcttagtattaattcggcaatc**TGA**ttctactcccccccagtacctgatactaccccccagtagattaatagtgctatgatttttcatattcttgttaaccaggtgttgccATGTCTCATACAATCCGTGATAAACAGAAACTGAAAGCGCGTGCC |
| *rhaB* | A**ATG**aaattcagcaggatcacattA**TGA**CCTTTCGCAATTGTGTCGCCGTCGATCTCGGCGCATCCAGT |
| *uvrY* | A**ATG**actaactatcag**TAG**cgttatccctatttctggagatattcctTTGATCAACGTTCTACTTGTTGATGACCACGAACTGGTGCGCGCA |
| *xap* | **ATG**tatttattatatggagcacttaattATGGAACGCGTATACAGAACAGATCT**TAA**GTTGCTCCGTTATTTT |
